# Potential Role of Mitochondria as Modulators of Blood Platelet Activation and Reactivity in Diabetes and Effect of Metformin on Blood Platelet Bioenergetics and Platelet Activation

**DOI:** 10.3390/ijms23073666

**Published:** 2022-03-27

**Authors:** Karolina Siewiera, Magdalena Labieniec-Watala, Hassan Kassassir, Nina Wolska, Dawid Polak, Cezary Watala

**Affiliations:** 1 Department of Haemostatic Disorders, Chair of Biomedical Sciences, Medical University of Lodz, 92-215 Lodz, Poland; hassan.kassassir1@gmail.com (H.K.); n.m.wolska@gmail.com (N.W.); dawidpolak1991@gmail.com (D.P.); cezary.watala@umed.lodz.pl (C.W.); 2 Department of Medical Biophysics, University of Lodz, 90-236 Lodz, Poland; magdalena.labieniec@biol.uni.lodz.pl; 3 Institute of Medical Biology, Polish Academy of Science, 93-232 Lodz, Poland; 4 Institute of Clinical Chemistry and Laboratory Medicine, University Medical Center Hamburg-Eppendorf, 20246 Hamburg, Germany

**Keywords:** blood platelets, diabetes mellitus, metformin, mitochondria, mitochondrial respiration, platelet activation, platelet reactivity

## Abstract

Blood platelet dysfunctions are strongly involved in the development of the micro- and macrovascular complications in diabetes mellitus (DM). However, the molecular causes of abnormal platelet activation in DM remain unclear. Experimental data suggests that platelet mitochondria can regulate the prothrombotic phenotype of platelets, and changes in these organelles may influence platelet activation and modify platelet responses to stimulation. The present study evaluates the impact of DM on mitochondrial respiratory parameters and blood platelet activation/reactivity in a rat model of experimental diabetes following 1, 2.5 and 5 months of streptozotocin (STZ)-induced diabetes. Moreover, a mild inhibition of the mitochondrial respiratory chain with the use of metformin under in vitro and in vivo conditions was tested as a method to reduce platelet activation and reactivity. The platelets were studied with a combination of flow cytometry and advanced respirometry. Our results indicate that prolonged exposure of blood platelets to high concentrations of glucose, as in diabetes, can result in elevated blood platelet mitochondrial respiration; this may be an effect of cell adaptation to the high availability of energy substrates. However, as these alterations occur later than the changes in platelet activation/reactivity, they may not constitute the major reason for abnormal platelet functioning in DM. Moreover, metformin was not able to inhibit platelet activation and reactivity under in vitro conditions despite causing a decrease in mitochondrial respiration. This indicates that the beneficial effect of metformin on the coagulation system observed in vivo can be related to other mechanisms than via the inhibition of platelet activation.

## 1. Introduction

Diabetes mellitus (DM) and uncontrolled hyperglycemia are well-recognized risk factors for the development of cardiovascular diseases, which are the major causes of death amongst patients with DM [1]. Diabetic patients are characterized by hyperactive platelets with exaggerated adhesion, aggregation and thrombin generation, and these features may also play a role in the development of cardiovascular events [2,3]. However, the mechanisms underlying elevated platelet activation and their hypersensitivity to stimulating factors in this condition remain obscure. Intensive changes in the physiology and morphology of these cells take place during platelet activation, and these require an increased amount of energy generated by glycolysis and oxidative phosphorylation. The key role played by energy in this process [4] makes mitochondria a potential regulator of platelet function, and therefore also a potential target for antiplatelet therapy. It seems highly probable that the prolonged exposure of blood platelets to high concentrations of glucose may lead to changes in the functioning of platelet mitochondria [5], especially considering the fact that this carbohydrate is an essential source of energy for these cells [6]. High concentrations of energy substrates may accelerate the work of mitochondria, leading to elevated oxygen consumption and increased mitochondrial membrane potential. This can support the accumulation of adenine nucleotides in platelets and thus regulate cytosolic levels of free calcium ions via platelet purinergic receptors [7]. The elevated substrate oxidation may also result in mitochondrial membrane hyperpolarization [5] and thus excessive reactive oxygen species (ROS) production, leading to indirect stimulation of the arachidonic acid metabolism by increasing phospholipase C activity; it can also have a direct influence by increasing the activity of prostaglandin H_2_ synthase. Either way, the process results in thromboxane A_2_ overproduction and intensified aggregation of hypersensitive platelets [8]. In addition to intracellularly produced ROS, diabetic blood platelets are constantly exposed to enhanced oxidative stress (OS) as a consequence of abundant glucose and products of its processing their environment [9].

Few studies have examined the relationship between the mitochondrial functioning and platelet activation, especially in the context of diabetes. There is rather a scarce body of evidence concerning mitochondrial respiratory chain activity in blood platelets from diabetic patients [10,11]; furthermore, such findings are also inconsistent and confusing in presenting either increased, reduced or unchanged functioning of mitochondria in this pathology. Therefore, the main aim of the present study was to evaluate whether changes in platelet activation/reactivity and changes in mitochondrial respiration occur at more or less the same moment during the development of diabetes, or independently each of other. The verification of this hypothesis could bring us closer to determining whether changes in mitochondria respiration can contribute to increased platelet activation and/or reactivity. For this purpose, both sets of parameters were measured in streptozotocin (STZ)-induced diabetic rats after 1, 2.5 and 5 months of confirmed, untreated diabetes. Moreover, assuming that mitochondrial respiratory activity is a preliminary and causative event in the pathway of platelet activation, it should be expected that a moderate inhibition of mitochondrial function (i.e., by the action of different drugs), understood as the mildly lowered ability to carry out oxidative phosphorylation, should result in a reduction in platelet activation and hypersensitivity. To test this hypothesis, metformin was selected for the study.

Metformin is a potent anti-hyperglycaemic drug that provides numerous beneficial side effects associated with the reduction in the micro- and macrovascular complications often observed in diabetic patients; including decreasing diabetes-related deaths, heart attacks and strokes [1]. However, the mechanism behind this beneficial effect remains poorly understood. In the context of our hypothesis, metformin is believed to act by inhibiting complex I of the mitochondrial respiratory chain of blood platelets, an effect demonstrated in in vitro studies [12]. Metformin treatment has been associated with a reduction in mitochondrial oxygen consumption and membrane potential in blood platelets, but mostly at concentrations exceeding those observed in plasma of patients treated with this drug [12]. In contrast, little is known of platelet activation in response to metformin. Therefore, our secondary aim was to evaluate the effect of metformin on blood platelet mitochondrial respiration, blood platelet activation and reactivity. For this purpose, a combination of in vitro and in vivo approaches was used. For the in vitro experiments, metformin was used at a wide range of concentrations corresponding to those observed in the plasma of diabetic patients treated with this drug (20 µM and 100 µM), concentrations noted in patients with drug-induced acidosis (500 µM), as well as much higher concentrations (1 mM and 5 mM). For the in vivo study, metformin was introduced at the dose of 50 mg/kg b.w./day to a rat model after 1 month of STZ-diabetes; the drug was then administered for 4 months to mimic the clinical state of fully developed diabetes in humans treated with metformin for the next 120 days. The effects of two differentiating factors, diabetes and metformin treatment on platelet activation, reactivity and mitochondrial respiration, were compared in four groups of animals: metformin-treated and untreated animals with or without diabetes.

## 2. Results

### 2.1. Effect of Metformin on Mitochondrial Respiration and Platelet Activation and Reactivity under In Vitro Conditions

Two-hour incubation of isolated platelets with metformin is sufficient to affect platelet mitochondrial respiration, but only at extremely high concentrations (Table 1). ROUTINE respiration (*R*, a physiological coupling state governed inter alia by cellular energy demand) and maximal electron system capacity (ET) were reduced for metformin used at 1 mM and 5 mM concentration (a detailed description of the measured parameters is given in Section 4.6.)

Changes in LEAK respiration (*L*, respiration related to the compensation of electron leak, proton leak, proton slip and cation cycling), changes in maximal electron system capacity measured after complex I (CI) inhibition by rotenone (ETrot) and changes in non-mitochondrial respiration (ROX) were not observed, even at the highest metformin concentration. A decreased ROUTINE control ratio (R/E, a parameter indicating the portion of the electron transfer capacity used during routine cell activity) was observed for platelets incubated with 1 mM or 5 mM of metformin. However, these differences were not statistically significant. The incubation of platelets with metformin at lower concentrations (0–500 μM) did not cause any direct changes in mitochondrial respiration parameters or in the calculated control ratios (Table 1).

**Figure 1:**
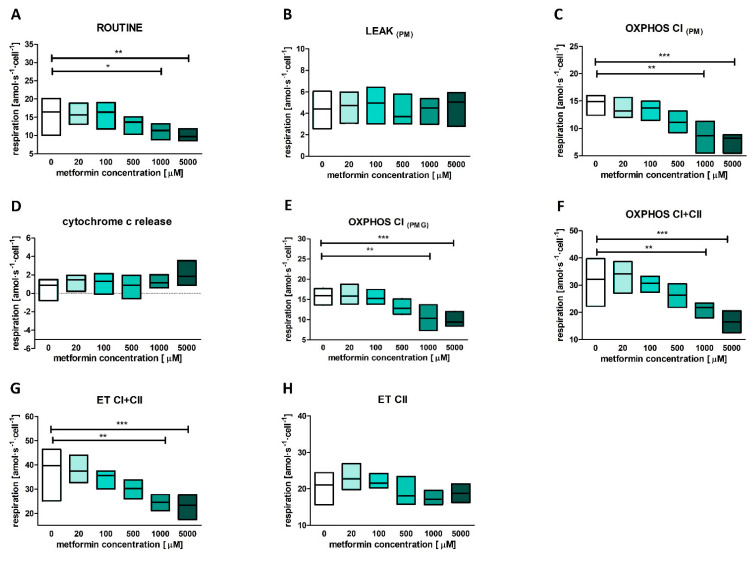
Effects of increasing metformin concentrations on mitochondrial respiration parameters measured for permeabilized blood platelets (**A**–**H**). Respiration was measured for ROUTINE state (**A**), LEAK state (**B**), OXPHOS supported by pyruvate and malate (**C**), OXPHOS supported by pyruvate, malate and glutamate (**E**), OXPHOS supported by CI+CII substrates (**F**), in the maximally noncoupled state to evaluate electron transfer capacity supported by CI+CII substrates (**G**), and to evaluate electron transfer capacity supported by CII substrates (**H**). The difference between respiration before and after addition cytochrome c was used to evaluate the integrity of outer mitochondrial membrane (**D**). Data presented as medians (solid line) and IQR (boxes) for control animals, *n* = 8. A more detailed description of the measured parameters is given in Section 4.6. The significances of the differences, as estimated with the repeated measures ANOVA and the post-hoc Dunnett’s multiple comparison test, were: * *p* < 0.05, ** *p* < 0.01, *** *p* < 0.001.

Decreased mitochondrial respiration was observed in permeabilized platelets under the influence of the two highest metformin concentrations and some moderately inhibitory, yet insignificant, effect was noted at 500 μM concentration of metformin (Figure 1). As seen for intact platelets, respiration was also reduced before the addition of digitonin. Moreover, mitochondrial respiration remained decreased when oxidative phosphorylation (OXPHOS) and maximal electron system capacity were measured. In the case of OXPHOS, the changes were observed both when complex I (CI) substrates were supplied and when a combination of CI and complex II (CII) substrates was used. However, maximal capacity was reduced only when mitochondria were supplemented with a combination of CI and CII substrates. All changes remained significant merely for metformin concentrations of 1 mM and 5 mM. No changes were observed in the case of LEAK respiration, cytochrome c release (marker of the outer mitochondrial membrane integrity) or maximal respiration supported by complex II substrates (Figure 1).

The effect of metformin on platelet activation and reactivity in response to in vitro stimulation was assessed after two-hour incubation. Metformin, regardless of its concentration, did not inhibit spontaneous activation of non-stimulated platelets during storage: P-selectin expression was 3.0 (2.2–4.3)% after platelet isolation, while the expression values after two-hour incubation are given in Table 2. The statistical significance of the differences between freshly-isolated platelets and those incubated for two-hours with 0 μM, 100 μM and 1000 μM of metformin was *p* < 0.01, and *p* < 0.001 for 20 μM, 500 μM and 5000 μM of metformin (ANOVA and post hoc Dunnett’s multiple comparison test). Metformin did not influence platelet activation in response to collagen, ADP or thrombin stimulation (Table 2).

### 2.2. Experimental Diabetes–Characteristics of Animal Groups

The animals with 1-month, 2.5-month and 5-month diabetes were characterized by reduced body weight, elevated non-fasting blood glucose levels and elevated content of glycated haemoglobin in comparison to the nondiabetic age-matched control rats (Table 3). Supplementation with metformin led in diabetic animals to slight, but significant, decrease in blood glucose level and HbA1c concentration compared to animals not receiving metformin. However, it did not affect the body weight of the rats regardless of the study group.

### 2.3. Platelet Activation and Reactivity in Control and Diabetic Animals

**Figure 2:**
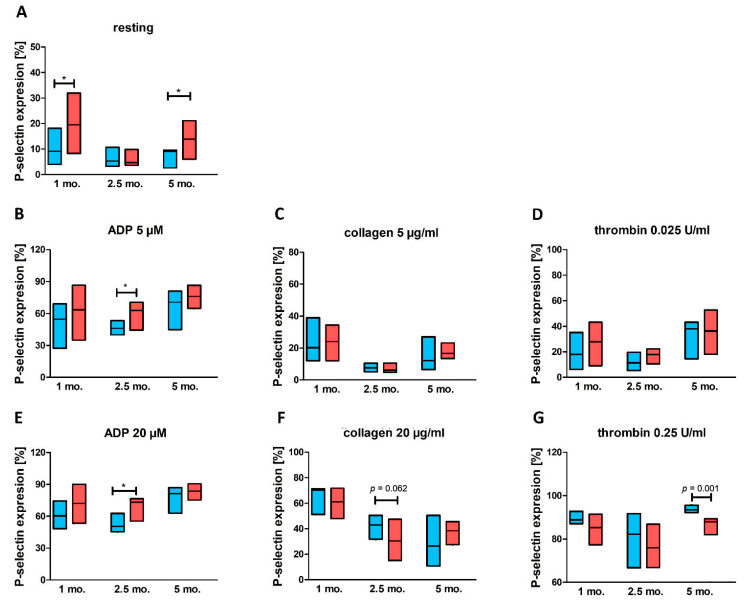
Activation and reactivity of platelets from diabetic animals and their age-matched non-diabetic controls (**A**–**G**). Data are presented as medians (solid line) and IQRs (boxes) for control animals (blue boxes) and diabetic animals (red boxes); *n* = 7–14. P-selectin expression was measured on the surface of resting (**A**) and agonist-stimulated platelets:ADP 5 µM (**B**) or 20 µM (**E**), collagen 5 µg/mL (**C**) or 20 µg/mL (**F**), thrombin 0.025 U/mL (**D**) or 0.25 U/mL (**G**), collected from rats with 1-month, 2.5-month and 5-month diabetes and their age-matched controls. The significances of the differences, as estimated with unpaired Student *t* test or Mann–Whitney *U* test, were: * *p* < 0.05.

A significant increase in the expression of P-selectin was already observed on the surface of platelets from STZ-diabetic animals in comparison to controls after 1 month of diabetes (Figure 2A). Interestingly, this was not observed after 2.5 months of diabetes, but returned after 5 months.

Platelet reactivity was tested in response to lower and higher concentrations of ADP (5 μM and 20 μM), collagen (5 μg/mL and 20 μg/mL) and thrombin (0.025 U/mL and 0.25 U/mL). In the case of lower agonist concentration, hyperreactivity of platelets from diabetic animals was observed only after ADP stimulation of platelets from 2.5-month diabetic animals (Figure 2B). No differences in platelet response was found between diabetic animals and age-matched controls stimulated with lower collagen (Figure 2C) or lower thrombin concentration (Figure 2D). Furthermore, no changes were observed between metformin-treated and untreated animals in response to these agonists at lower concentrations (Figure 2C,D). Following the stimulation with 20 μM ADP, platelet hyperreactivity (elevated response) was observed among diabetic rats in comparison to the age-matched control group, but only for the 2.5-month group (Figure 2E). In contrast, in the 2.5-month group, lower P-selectin expression (platelet hyporeactivity) was noted in diabetic rats compared to non-diabetic animals after platelet stimulation with 20 μg/mL collagen (Figure 2F). This effect was not observed for other studied groups of animals in high collagen-stimulated platelets. However, platelet hyporeactivity in response to 0.25 U/mL thrombin was also noted for 5-month diabetic rats when compared to the age-matched control group (Figure 2G).

### 2.4. The Impact of the Diabetes on Platelet Mitochondrial Respiration—Protocol for Intact Platelets

**Figure 3:**
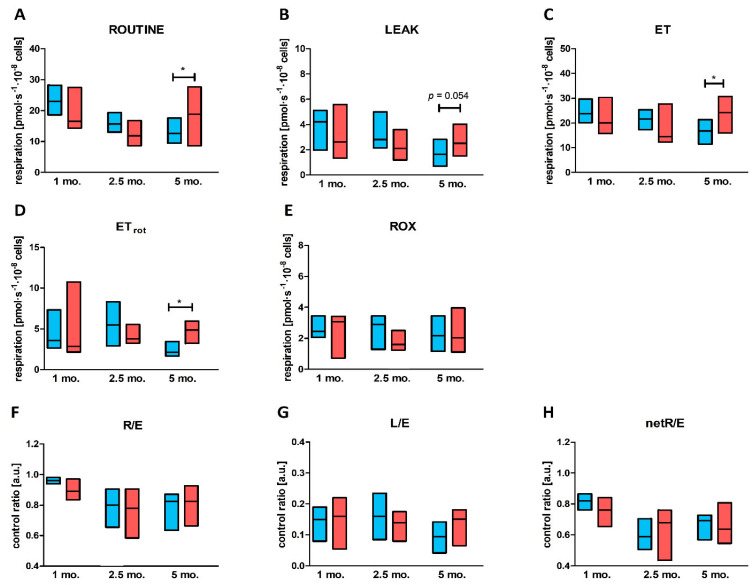
Mitochondrial respiration parameters, control ratios and non-mitochondrial respiration measured for intact blood platelets obtained from diabetic animals and their age-matched controls. Respiration was measured for ROUTINE state (**A**), LEAK state (**B**), in the maximally noncoupled state to evaluate electron transfer capacity (**C**), and to evaluate electron transfer capacity after CI inhibition (**D**), and after inhibition of electron transfer to evaluate (residual) non-mitochondrial respiration component (**E**). In addition, ratio of respiration for ROUTINE state to ET capacity, R/E (**F**), ratio of respiration in LEAK state to ET capacity, L/E (**G**), and netROUTINE control ratio indicating the proportion of total electron transfer capacity used for oxidative phosphorylation (**H**) were calculated. Data are presented as medians (horizontal line) and IQRs (boxes) for control animals (blue boxes) and diabetic animals (red boxes); *n* = 9–14. All values recorded for the states of ROUTINE, LEAK, ET and ETrot capacity were corrected for ROX. For a more detailed description of the measured parameters, please refer to Section 4.6. The significances of the differences, as estimated with unpaired Student *t* test or Mann–Whitney *U* test, were: * *p* < 0.05.

No changes in intact platelet mitochondrial respiration (ROUTINE, LEAK, ET, ETrot), control ratios (*R/E*, *L/E,* net*R/E*) or non-mitochondrial respiration (ROX) were observed between diabetic animals and age-matched non-diabetic controls after 1 and 2.5-months of confirmed diabetes. However, after 5 months, platelet mitochondrial respiration was found to increase among diabetic animals in comparison to age-matched controls. ROUTINE respiration and LEAK respiration were elevated, but the differences were statistically significant only for ROUTINE respiration (Figure 3A,B). The maximal electron transfer system capacity (ET capacity) and ET capacity after rotenone addition (ETrot) were also elevated for platelets from diabetic animals (Figure 3C,D). Noncoupled respiration after complex I inhibition was significantly elevated for platelets from diabetic animals in comparison to age-matched controls. All control ratios (*R/E*, *L/E,* net*R/E)* remained unchanged between 5-month diabetic rats and control animals (Figure 3F–H). No changes in the levels of non-mitochondrial respiration were noted (Figure 3E).

### 2.5. The Impact of Long-Lasting Diabetes and Metformin Treatment on Platelet Activation and Mitochondrial Respiration

For multiple comparisons when four groups were compared (diabetic, control and both groups of animals treated with metformin), differences in resting activation observed for 5-month diabetic animals vs. control rats were at the border of statistical significance (*p* = 0.057, Table 4). However, hyporeactivity of diabetic platelets in response to higher thrombin level was still observed. The four months of supplementation with metformin had no impact on platelet activation in the diabetic group nor in the control group. Interestingly, platelets from 5-month diabetic animals treated with metformin responded to high thrombin concentration at the same level as platelets from non-diabetic control animals and non-diabetic control animals treated with metformin (Table 4).

**Figure 4:**
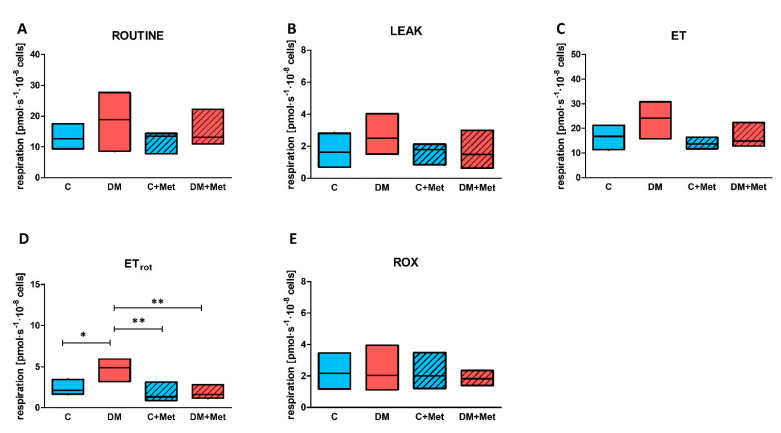
Mitochondrial respiration parameters and control ratios measured for intact blood platelets obtained from rats with 5-month diabetes and their age-matched controls, as well as in control and 5-month diabetic rats treated with metformin. Respiration was measured for ROUTINE state (**A**), LEAK state (**B**), in the maximally noncoupled state to evaluate electron transfer capacity (**C**), and to evaluate electron transfer capacity after CI inhibition (**D**), and after inhibition of electron transfer to evaluate (residual) non-mitochondrial respiration component (**E**). All values recorded for the states of ROUTINE, LEAK, OXPHOS and ET were corrected for ROX. Data are presented as medians (horizontal line) and IQRs (boxes) for control animals (blue boxes), diabetic animals (red boxes) and animals treated with metformin (blue and red boxes, respectively, with diagonal lines); *n* = 11–14. For a more detailed description of the measured parameters, please refer to Section 4.6. The significances of the differences, as estimated with the Kruskal–Wallis test and the Conover–Iman post hoc test for multiple comparisons, were: * *p* < 0.05, ** *p* < 0.01.

For multiple comparisons when four groups were compared (diabetic, control and both groups of animals treated with metformin) differences in ROUTINE respiration and ET capacity observed for 5-month diabetic animals vs. control rats in intact platelets (Figure 3A,C) did not reach statistical significance (Figure 4A,C). However, elevated ET capacity after complex I inhibition (ETrot) in DM group remained significantly increased in comparison to control group (Figure 4D).

**Figure 5:**
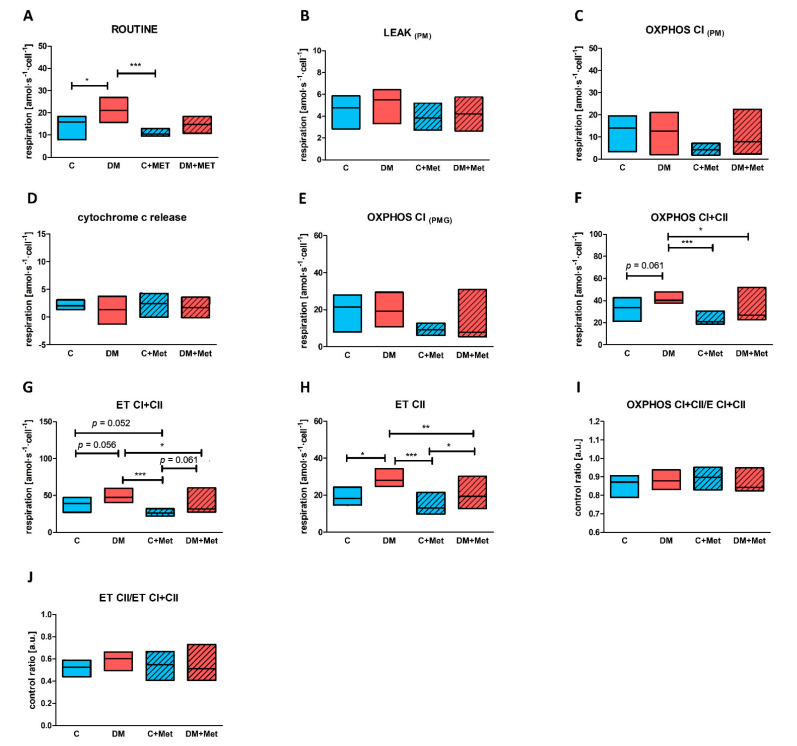
Mitochondrial respiration parameters and control ratios measured for permeabilized blood platelets obtained from rats with 5-month diabetes and their age-matched controls, as well as in control and 5-month diabetic rats treated with metformin (**A–J**). Respiration was measured for ROUTINE state (**A**), LEAK state (**B**), OXPHOS supported by pyruvate and malate (**C**), OXPHOS supported by pyruvate, malate and glutamate (**E**), OXPHOS supported by CI+CII substrates (**F**), in the maximally noncoupled state to evaluate electron transfer capacity supported by CI+CII substrates (**G**), and to evaluate electron transfer capacity supported by CII substrates (**H**). The difference between respiration before and after addition cytochrome c was used to evaluate the integrity of outer mitochondrial membrane (**D**). In addition, ratio of respiration for OXPHOS CI+CII to ET capacity supported by CI+CII (**I**), and ratio of respiration measured for ET capacity supported by CII to ET capacity supported by CI+CII (**J**) were calculated. Data are presented as medians (horizontal line) and IQRs (boxes) for control animals (blue boxes), diabetic animals (red boxes) and animals treated with metformin (blue and red boxes, respectively, with diagonal lines); *n* = 8–14. Oxygen consumption of permeabilized platelets collected from 5-month diabetic rats and their age-matched controls, as well as 5-month diabetic and control rats treated with metformin. All values recorded for the states of ROUTINE, LEAK, OXPHOS and ET were corrected for ROX. For a more detailed description of the measured parameters, please refer to the Section 4.6. The significances of the differences, as estimated with the Kruskal–Wallis test and the Conover–Iman post hoc test for multiple comparisons, were: * *p* < 0.05, ** *p* < 0.01, *** *p* < 0.001.

Routine oxygen consumption before permeabilization was similar to that obtained in the intact platelets protocol and was significantly higher for diabetic animals than for controls (Figure 5A). Similar rates of mitochondrial respiration linked with CI substrates were observed for all groups (LEAK_PM_) (Figure 5B). No differences were found in respiration coupled with ATP production supported by pyruvate and malate—CI-linked oxygen phosphorylation (OXPHOS CI_PM_) (Figure 5C). All groups demonstrated similar differences in respiration before and after the addition of cytochrome c; this indicates no differences in the integrity of outer mitochondrial membrane between these groups (Figure 5D). Oxygen phosphorylation supported by the combination of pyruvate, malate and glutamate also did not differ between the studied groups (OXPHOS CI_PMG_) (Figure 5E). The diabetic animals demonstrated a slight, but not significant, elevation in respiration when oxygen phosphorylation was supported by a combination of CI and CII substrates (OXPHOS CI+CII) (Figure 5F) and in maximal oxidative capacity (ET CI+CII) measured after uncoupler titration (Figure 5G). Noncoupled mitochondrial respiration after the inhibition of CI with rotenone (ET CII) was significantly higher for the diabetic group in comparison to control animals (Figure 5H). However, platelets from diabetic animals demonstrated no increase in ET CII/ET CI+CII ratio in comparison to controls (Figure 5J), indicating that both groups have similar substrate utilization profiles. Oxidative phosphorylation for CI+CII was about 10–16% lower than maximal respiratory capacity for CI+II by indicating a slight flux limitation system at saturating concentrations of exogenous substrates; however, its level is similar for all tested groups (Figure 5I).

Supplementation with metformin had no significant effect on ROUTINE respiration. Similarly, LEAK respiration remained unchanged in metformin-treated animals in comparison to untreated animals (Figure 5B). Additionally, no changes in the outer membrane integrity (cytochrome c release) were observed following metformin treatment (Figure 5D). Interestingly, metformin had no impact on oxidative phosphorylation supported by CI substrates (OXPHOS CI_PM_ and OXPHOS CI_PMG_); however, the median of mitochondrial respiration values in these cases was slightly lowered for platelets obtained from animals treated with metformin (C+Met and DM+Met) in comparison to the untreated groups (C and DM) (Figure 5C,E). Decreased mitochondrial respiration was observed for oxidative phosphorylation supported by combination of CI and CII substrates in the case of diabetic animals treated with metformin compared to untreated diabetic animals (Figure 5F). Maximal oxidative capacity (ET CI+CII) was also slightly, but significantly, lower for the diabetic group treated with metformin compared to the untreated diabetic group (Figure 5G). These changes were not observed between control animals and control animals treated with metformin for either ET CI+CII or OXPHOS CI+CII (Figure 5F,G). Interestingly, metformin supplementation led to the decreased noncoupled respiration after complex I inhibition (ETrot in case of intact platelets and ET CII in case of permeabilized blood platelets) in metformin-treated diabetic animals vs. untreated diabetic animals (Figure 4D and Figure 5H). Such changes were not observed for control animals treated with metformin vs. untreated control animals (Figure 4D and Figure 5H).

## 3. Discussion

Diabetes is associated with an elevated predisposition to cardiovascular complications [1]. These complications are related to various haemostatic abnormalities such as impaired fibrinolysis, endothelial dysfunction, increased activity of coagulation factors, increased platelet adhesiveness, exaggerated aggregation and platelet hyperreactivity [13,14]. Diabetic patients and animals typically demonstrate elevated platelet activation and changes in platelet response to stimulation, including hyperreactivity to weak or low concentrations of agonists and hyporeactivity to strong or high concentrations of agonists [2,13,15]. Such changes, together with changes occurring in platelet mitochondrial respiration, have been noted in diabetic rats in our previous study; the analysis also showed a positive correlation between P-selectin expression and ROUTINE respiration, and between P-selectin expression and electron transfer capacity when diabetic non-stimulated platelets were taken into account [5]. A similar observation was also made by Villarroel et al., indicating a strong positive correlation between platelet mitochondrial respiration and the propensity of platelets toward activation [16]. Therefore, the present study evaluates whether changes in mitochondrial respiration may result in altered platelet activation and reactivity profile in diabetic animals. For this purpose, we checked whether the development of these changes occurs simultaneously in the course of diabetes or whether one of these changes appears first and then determines the presence of the other one.

Our results show that the profile of platelet activation and reactivity may be changed depending on the duration of diabetes, with platelets from STZ-injected animals demonstrating a higher activation state than controls even after the first month of diabetes. In addition, P-selectin expression was found to be slightly elevated in response to both tested concentrations of ADP after the first month of diabetes, but these differences were not statistically significant. After 2.5 months of diabetes, certain differences in platelet reactivity were noted between these groups: hyperreactivity of diabetic platelets to ADP simulation and the trend of lowered response of diabetic platelets to higher concentration of collagen. However, no changes in mitochondrial respiration parameters and control ratios were found between diabetic animals and their age-matched control group for these time points. Only after 5 months did the changes in platelet activation/reactivity profile and the changes in mitochondrial functioning appear to proceed in parallel. The diabetic animals demonstrated elevated activation and hyporeactivity of platelets following stimulation with higher concentrations of thrombin and greater platelet mitochondrial respiration in most respiratory states. Therefore, our results indicate that the prolonged exposure of blood platelets to high concentrations of glucose in diabetes can lead to changes in the functioning of blood platelet mitochondria. It is noteworthy, however, that all these alterations in the respiration occur later than the changes in platelet activation and reactivity. This clearly shows that platelet activation and reactivity are modulated from the onset of diabetes and differ from that observed for normoglycemic animals. Otherwise, the changes in mitochondrial respiration between these two groups were not seen until the fifth month of diabetes, and hence they cannot be regarded as the leading cause of abnormal platelet functioning revealed at earlier stages of DM. This is the first such observation published to date.

Changes in mitochondrial respiration observed for diabetic animals after 5 months of diabetes were similar to those observed in our previous study [5], in which mitochondrial respiration was found to be elevated in all the measured parameters (ROUTINE, LEAK and ET capacity) for intact diabetic platelets compared to control rats, but no changes in control ratios were observed. Therefore, these alterations were most probably related to the increase in mitochondrial mass, indicated by the significantly elevated amounts of cytochrome c oxidase observed in platelets from DM rats [5]. In the present study, the diabetic animals demonstrated elevated physiological coupling state respiration and proportionally greater electron transfer capacity. However, again, respiration control ratios did not differ between control and diabetic animals. Interestingly, maximal respiration induced by titration of protonophore and registered after the inhibition of complex I was significantly elevated in intact platelets from diabetic animals (ET_rot_), which indicates elevated respiration related to CII substrates. Oxidative phosphorylation supported by CI+CII substrates as well as maximal electron capacity supported by CI+CII were also slightly elevated in the case of diabetic platelets. However, this increase was not statistically significant, in contrast to the elevated electron capacity supported by CII substrates. This could suggest increased energy production supported by succinate. However, the lack of differences in ET CII/ET CI+CII ratio between both groups of animals does not support this reasoning. These results are in contradiction with the data provided by Avila et al. [11], who noted that platelets obtained from type 2 diabetic patients exhibited decreased basal (ROUTINE) respiration, reduced electron transfer capacity and lowered oxidative phosphorylation [11]. Other publications report no effect of diabetes on platelet mitochondrial respiration [10,17]. Therefore, it is important to emphasize that the scientific literature concerning alteration of platelet mitochondrial respiration/functioning in diabetes is not only scarce, but also inconsistent. Moreover, according to our current results, these changes appear some time after the onset of diabetes, under conditions of prolonged uncontrolled hyperglycemia. This fact may also explain the discrepancy between different groups of researchers regarding the extent of platelet mitochondrial changes occurring in diabetes.

Along with the hypothesis that changes in the functioning of mitochondria in diabetes can modulate the activation and reactivity of platelets, the study proposed that a slight reduction in the activity of the mitochondrial chain can yield beneficial effects by alleviating the increased activation of diabetic platelets and their hypersensitivity in response to stimulation with physiological agonists. 

The most commonly used antidiabetic drug, metformin, is known to have beneficial effects on reducing cardiovascular complications in diabetic patients [1]. However, the mechanisms behind this effect remain unclear, and only a few studies have examined the impact of metformin on blood platelets. Since one of the properties of metformin is its ability to inhibit mitochondrial complex I, regarded as one of disadvantages of this drug [18], it should be able to reduce platelet activation via attenuating mitochondrial respiration; this would paradoxically improve the platelet response profile to activating factors. In the present study, no changes in mitochondrial respiration were observed at metformin concentrations corresponding to those observed in serum/plasma of patients treated with this drug (up to 100 μM), or even a few times higher concentrations, noted in serum/plasma of patients with a metformin-related acidosis, 500 μM. However, the platelets treated with 1 mM and 5 mM metformin demonstrated decreased ROUTINE (physiological) respiration, together with a drop in oxidative phosphorylation supported by CI substrates and by CI+CII substrates and a decreased maximal electron capacity (ET) supported with CI+CII substrates, although no decreased activation of non-stimulated (resting) platelets was seen nor was any reduction in platelet response to agonist stimulation, for either metformin concentration in vitro.

Our results are in line with those of Protti et al., who found that metformin has a dose- and time-dependent effect in human platelet mitochondria [12]. The authors showed that metformin can cause mitochondrial dysfunction including decrease in mitochondrial complex I activity, mitochondrial membrane potential and oxygen consumption, and lead to lactate overproduction in human platelets incubated in vitro for 72 h [12]. However, most of these effects were not observed at concentrations below 1 mM, and some of them, specifically platelet complex I inhibition and decrease in mitochondrial membrane potential, were noted only at the extremely high concentration of 100 mM [12]. Interestingly, the decrease in oxygen consumption (corresponding to ROUTINE respiration) was already observed for 10 μM metformin [12]. Probably, the effects of such a long incubation period could drastically reduce platelet mitochondrial respiration, as observed elsewhere [19], and at the same time increase the impact of metformin on mitochondrial electron transfer system, and thus also on mitochondrial respiration. In contrary to us, no platelet activation parameters were assessed by Protti et al. [12]. Similar observations have been made by Piel and colleagues regarding human platelet complex I inhibition under in vitro conditions [18], who also found that metformin demonstrated a dose- and time-dependent respiratory inhibition leading to significantly decreased ROUTINE respiration and decreased maximal uncoupled respiration following the exposure to 100 mM metformin [18]. In addition, similar to our study, platelet incubation with 1 mM metformin resulted in decreased oxidative phosphorylation (OXPHOS) capacity for CI and a similar decrease in OXPHOS capacity for CI+CII and electron transfer (ET) capacity for CI+CII. The ET capacity for CII substrates, the activity of complex IV and the CI+CII-mediated LEAK respiration were not changed in the presence of metformin [18]. Similarly, no significant changes in LEAK respiration or ET CII were observed in the present study. Unfortunately, no platelet activation parameters were evaluated in the study of Piel et al. [18]. In contrast, a study by Xin et al. is one of the very few examples assessing the effect of metformin on both platelet mitochondria and platelet activation. Authors confirmed that six-hour incubation of platelets with 1 mM metformin inhibits activity of complex I [20]. However, unlike our results, they showed that metformin decreased platelet in vitro response to ADP (measured by the expressions of P-selectin and the activated α_IIb_β_3_ complex). They also observed that metformin significantly decreased platelet aggregation induced by ADP, arachidonic acid or thrombin and inhibited platelet adhesion to collagen-coated surface [20]. However, their study used a much longer incubation time. Since long-term storage adversely affects mitochondrial respiration and leads to artefactual platelet activation [19], a shorter incubation period was used in our present study. There are also several reports in the literature on the effect of metformin in vitro on other processes related to platelet functioning, such as platelet adhesion [21], platelet aggregation [21,22], thrombus formation [23] or thromboxane production [22]. However, these findings are also inconsistent in terms of either inhibitory effect [21,22] or no effect [21,22,23] of this drug on these pro-thrombotic processes. 

In vivo and ex vivo experiments evaluating the effect of metformin have provided a valuable information on whether the concentrations obtained in a living organism may actually have an influence on blood platelets. Protti et al. report decreased activity of complex I and complex IV in platelets obtained from human patients suffering from the metformin-related lactic acidosis (serum drug level 192 ± 84 μM) [12]. No such effect was observed in our in vitro study, even at five times higher metformin concentration; however, it is worth mentioning that both studies used different methods of assessing the functioning of the tested mitochondrial complexes, and the short in vitro incubation, even with a higher concentration of metformin, cannot replicate the effects of lower plasma concentrations of a drug acting for much longer period [12]. In our study, supplementation with 50 mg/kg/day metformin did not statistically reduce ROUTINE platelet mitochondrial respiration, as observed in our in vitro experiment, although the median ROUTINE values were lower in animals receiving metformin. It also had no impact on the dissipative component of respiration (LEAK) and integrity of outer mitochondrial membrane. No inhibition of respiration related to oxidative phosphorylation supported by complex I substrates was observed. However, our findings indicate lowered OXPHOS respiration and ETS capacity for the combination of complex I and II substrates in diabetic animals receiving metformin in comparison to non-treated diabetic animals. This could be the effect of the higher respiration level that would potentially allow detection of more subtle differences. However, what is more probable, especially in the context of the whole tested period, is that metformin treatment was able to reduce the impact of diabetes on mitochondria and to prevent diabetic platelets from the changes in mitochondrial function being developed between 2.5 and 5 months of diabetes. This effect was not related to the inhibition of complex I of mitochondrial electron transfer system, as demonstrated in the in vitro part of our study. In our present study, metformin supplementation decreased neither the activation nor reactivity of platelets. Interestingly, it appears that metformin treatment could restore the ability of diabetic platelets to respond to stimulation with a strong agonist, as evidenced for diabetic platelets stimulated with 0.25 U/mL thrombin. This is in line with the observation that metformin is able to protect platelet mitochondria against diabetes-related changes occurring between 2.5 and 5 months of diabetes mellitus. This is, to the best of our knowledge, the first report presenting such data.

In contrast to our observations, Xin et al. report that seven-day administration of 400 mg/kg/day metformin to rats decreased platelet surface expression of P-selectin and the activated α_IIb_β_3_ complex following ADP stimulation, inhibited platelet adhesion to a collagen-coated surface and reduced ADP-, arachidonic acid- or thrombin-induced platelet aggregation [20]. However, these authors used a much higher dose of metformin compared to that applied in our present study [20]. An inhibitory effect of metformin on pro-thrombotic processes has also been observed in diabetic patients treated with metformin [24,25] with respect to reduced urinary 11-dehydro-thromboxane B_2_ level or with respect to reduced ADP-induced platelet aggregation [26]. Other publications report no effect of metformin on the functioning of platelets [27,28], as in the case of diabetic patients treated with dual antiplatelet therapy (DAPT) in combination with various antidiabetic drugs (insulin or metformin). No differences were observed in platelet activation and reactivity, platelet aggregation nor in thromboxane B_2_ (TXB_2_) level, regardless of whether the patients were treated with insulin or with metformin [28]. There were also no significant effects of metformin on platelet β-thromboglobulin or platelet factor 4 concentrations on platelet spontaneous and ADP-induced aggregation among non-insulin-dependent diabetic patients treated for 12 weeks with this drug [27]. This highlights that very little is yet known about the effect of metformin on the platelet pro-thrombotic processes, including platelet activation, and about the effect of mitochondrial inhibition on platelet functioning in general.

Nevertheless, our results indicate that metformin may prevent or at least delay the development of unfavorable changes in the course of diabetes. Similar observations have been made by Rösen et al. in the context of carbonyl concentration, level of tissue lipid peroxides and the loss in aconitase activity associated with the development of diabetes in Goto-Kakizaki rats treated with metformin [29]. The ability of metformin to delay the appearance of different types of pathological changes was also observed in the case of atherosclerosis in T1DM [30], nephropathy in STZ-diabetic rats [31], endothelial dysfunction under in vitro conditions of fluctuating glucose [32] and cardiac injury after acute coronary syndrome in diabetic patients [33]. Likewise, slight reduction or delay in the development of diabetes in patients at the increased risk of this disease was concluded by Madsen et al. from the meta-analysis performed for 20 randomized controlled trials with a total number of 6774 participants [34].

In conclusion, our results indicate that prolonged exposure of blood platelets to high concentrations of glucose in diabetes can lead to the elevated blood platelet mitochondria respiration. However, these alterations occur later than the changes in blood platelet activation/reactivity. Thus, they may not constitute the major contributor to abnormal platelet functioning in diabetes mellitus. In vivo administration of metformin to diabetic animals with already developed diabetes can impact mitochondria without the effect on the complex I activity. We conclude that metformin was able to prevent diabetic platelets from the changes in mitochondrial function developing between 2.5 months and 5 months of diabetes. However, metformin treatment has not been demonstrated to reduce the activation or restrain the hypersensitivity of diabetic platelets to agonist stimulation. On the contrary, it was found to restore the ability of diabetic platelets to respond to some stimulation agents (such as thrombin) to a similar extent, as in the case of platelets from untreated healthy (control) animals. Moreover, the in vitro study showed that metformin appears to influence platelet mitochondrial respiration at concentrations greater than 1 mM. However, no parallel changes in platelet activation were noted, thus indicating that either metformin has no apparent impact on platelet activation at the tested concentrations or that the incubation time was insufficient. Therefore, all these findings lead to the conclusion that the preventive effect of metformin in relation to cardiovascular complication in diabetic patients is most probably mediated through different mechanism(s) than the reduced platelet response to activating factors.

Study limitations: A wide range of metformin concentrations (50–500 mg/kg/day) have been used in in vivo studies assessing the effects of metformin in laboratory rat models of diabetes [35,36,37,38,39]. However, only two doses were used to evaluate the impact of this drug on pro-aggregatory function of platelets: 400 mg/kg/day for 7 days was used to evaluate the impact of metformin on platelet aggregation [20] and 300 mg/kg/day for 15 days to evaluate its impact on vascular function in diabetic rats [40]. Since longer periods of metformin administration were used in our study than those reported previously, the drug concentration was deliberately reduced to 50 mg/kg/day. This dose was found to be insufficient for decreasing glycation parameters to the expected levels and was also too low to inhibit mitochondrial complex I. Regardless, the applied dose inhibited the changes in mitochondrial respiration resulting from diabetes progression and restored the ability of platelets to respond to strong agonists, reaching the levels of their response observed for platelets from control rats. Nevertheless, it seems that higher doses of metformin should be tested in future in vivo experiments when evaluating metformin impact on platelet activation.

## 4. Materials and Methods

### 4.1. Chemicals 

The sodium citrate used as an anticoagulant was from Becton-Dickinson (Franklin Lakes, NJ, USA). FITC-labelled anti-GPIIIa gating antibodies were from Novus (Centennial, CO, USA). PE-labelled anti-P-selectin antibodies and isotype control were from eBioscience (San Diego, CA, USA). Metformin hydrochloride was from Polpharma (Starogard Gdanski, Poland). All material and chemicals used in the course of this study were obtained from Sigma (St. Louis, MO, USA), unless otherwise stated. Ketamine and xylazine for animal anaesthesia were purchased from Biowet-Pulawy (Pulawy, Poland). Phosphate buffered saline (PBS) was from Corning (New York, NY, USA). Water used for the experiments (chemicals and buffers preparation, glassware washing) was purified using Easy Pure UF unit (Thermolyne Barnstead, Dubuque, IO, USA).

### 4.2. Animals

For in vitro study, 16 Sprague–Dawley control (non-diabetic) rats (males, 460–540 g) were anaesthetized with ketamine (100 mg/kg b.w.) and xylazine (23.32 mg/kg b.w.) and blood was collected from the abdominal aorta into a tube containing 3.2% sodium citrate. Blood platelets were isolated and the impact of metformin on respiration and activation was tested. For the part of in vivo study, 110 8-week-old Sprague–Dawley rats (males, 180–300 g) were randomly, non-proportionally allocated into two groups: control (non-diabetic) animals (45 rats) and diabetic animals (65 rats). In order to induce diabetes, the animals were intraperitoneally injected with streptozotocin (STZ) dissolved in 0.1 mol/L citrate buffer, pH 4.5, at the dose of 65 mg/kg of body weight. The control group of animals received a vehicle (citrate buffer). STZ-injected animals with blood glucose level higher than 300 mg/dL after seven days from the injection were considered diabetic and included into the group of diabetic animals. After 1 month from the beginning of the study, in selected cohorts of control (14 rats) and diabetic animals (21 rats), metformin treatment was commenced at 50 mg/kg b.w./day. The drug was dissolved freshly from a powder every day into the drinking water, taking into account the rat body weight (measured weekly) and the volume of water that animals consume. Animals not treated with metformin received pure drinking water during this period. If the water with metformin was entirely consumed, the bottle was replaced with normal water. Treatment continued for four months until the end of the experiment. After 1, 2.5 and 5 months, the animals that survived the treatment were anaesthetized with ketamine (100 mg/kg b.w.) and xylazine (23.32 mg/kg b.w.) and their blood was collected from the abdominal aorta into a tube containing 3.2% sodium citrate. In the course of this study, the following groups of animals were examined:

a) STZ animals with 1-month diabetes and their age-matched control group;
b) STZ animals with 2.5-month diabetes and their age-matched control group;
c) STZ animals with 5-month diabetes and their age matched control group, STZ animals with 5-month diabetes treated for four months with metformin at the dose of 50 mg/kg b.w./day and their age matched control group treated for four months with metformin at the dose of 50 mg/kg b.w./day.

During the experiment, the animals were housed under standard environmental conditions (25 °C, with a light/dark cycle of 12 h/12 h) with a continuous access to food (standard chow) and water. The animals were under constant veterinary supervision: routine veterinary inspections assessing the overall welfare of the animals were carried out daily. The experiments were conducted in accordance with the US National Institute of Health and the EU Directive 2010/63/EU for animal experiments and approved by the Local Ethical Committee on Animal Experiments at the Medical University of Lodz (13/LB603/DLZ/2017 of 6 February 2017 and 13/LB18/2017 of 8 May 2017).

### 4.3. Blood Collection and Sample Preparation

Sampling of whole blood was performed in the morning after overnight fasting. Blood was collected from the abdominal aorta into a laboratory tube with 3.2% sodium citrate. Blood platelets were isolated immediately after blood collection. For this purpose, blood was diluted 1:1 with warm PBS (37 °C) supplemented with 62.5 ng/mL prostaglandin E_1_ and centrifuged (190 g with no break, 12 min, 37 °C). The upper layer (diluted platelet-rich plasma) was transferred to a new tube and centrifuged (800 g with soft stop, 15 min, 37 °C). Plasma above the platelet pellet was removed. Next, in the case of in vitro experiments, the platelet pellet was suspended in a small amount of autologous platelet-poor plasma (PPP) and transferred to (a) Tyrode’s buffer (134 mM NaCl, 0.34 mM Na2HPO4, 2.9 mM KCl, 12 mM NaHCO3, 20 mM HEPES, pH 7.0, 5 mM glucose, 0.35% w/v BSA) for intact cell measurement or (b) MiR05 (110 mM sucrose, 60 mM K-lactobionate, 0.5 mM EGTA, 3 mM MgCl_2_, 20 mM taurine, 10 mM KH_2_PO_4_, 20 mM HEPES, pH 7.1 at 30 °C and 0.1% w/v BSA essentially fatty acid free) for permeabilized cell measurement. Samples were prepared to achieve a suspension of 1 × 10^8^ platelets/mL. The final PPP concentration in a suspension was up to 5%. In the case of in vivo experiments, the platelet pellet was additionally washed with Tyrode’s buffer with 3.2% sodium citrate (9:1, vol:vol) and 62.5 ng/mL prostaglandin E_1,_ and centrifuged again in the same conditions. PPP was prepared by the centrifugation of a small volume of whole blood (0.5 mL) at 2600 g for 15 min at room temperature and wormed up to 37° C after centrifugation. 

### 4.4. Morphological and Biochemical Measurements

Platelet count and contamination of samples with other blood cells were assessed using a IDEXX ProCyte Dx blood counter (IDEXX Laboratories Ltd., Windsor, UK). Blood glucose was assessed using Accu-Check glucometer (Roche Diagnostics Polska Ltd., Warsaw, Poland) and the content of glycated haemoglobin (HbA1c) was measured using an Olympus AV640 biochemical analyser (Olympus, Tokyo, Japan).

### 4.5. Flow Cytometric Analysis of Platelet Activation and Reactivity

To analyse the activation of circulating rat platelets, as well as their in vitro response to stimulation, the surface membrane P-selectin expression was monitored. For this purpose, the suspension of isolated platelets in Tyrode’s buffer prepared for respirometric measurements was supplemented with 1 mM CaCl_2_ and stained with PE-labelled anti-P-selectin antibodies and FITC-labelled anti-GPIIIa gating antibodies. Flow cytometric detection of platelet surface membrane antigens was performed within 30 min after staining on unfixed cells using the FACS Canto II instrument (Becton-Dickinson, Franklin Lakes, NJ, USA). Ten thousand GPIIIa/FITC-positive events were gathered. The percent of specific fluorescence-positive platelets was corrected by the subtracting of the non-specific isotype rat IgG1 binding. Reactivity was investigated after platelet stimulation with collagen (5 μg/mL or 20 μg/mL), ADP (5 μM or 20 μM) or thrombin (0.025 U/mL or 0.25 U/mL) for 15 min at RT.

### 4.6. Measurement of Mitochondrial Respiration

Mitochondrial respiration was recorded by a continuous measurement of oxygen consumption with a high-resolution respirometer equipped with a Clark-type oxygen sensor (Oxygraph-2k, Oroboros Instruments, Innsbruck, Austria). Experiments were performed at 37 °C in 2 mL chambers, with the stirrer speed set to 750 rpm and data recording interval of 2 s. Calibration at air saturation in respiration media (Tyrode’s buffer for intact blood platelets or MiR05 for permeabilized platelets) was conducted every day before the experiment started.

To test mitochondrial respiration, platelets were resuspended in Tyrode’s buffer (intact platelets) or MiR05 (permeabilized platelets) to obtain a concentration of 1 × 10^8^ platelets/mL. Mitochondrial respiration was tested in real-time by applying appropriate substrate–uncoupler–inhibitor titration (SUIT) protocols during the experiment. 

Measurements of intact platelets was performed according to Gnaiger and Renner-Sattler [41]: first, the platelet oxygen consumption was allowed to stabilize, and the respiration was captured in a physiological coupling state (ROUTINE, *R*)*;* oligomycin was then added (0.25 µM) to evaluate the dissipative component of respiration (LEAK respiration, *L*); next, the maximum electron transfer capacity (ET capacity, *E*) was measured by titration with the 0.25 µM steps of carbonyl cyanide p-trifluoromethoxyphenylhydrazone (FCCP) until no further increase in respiration was observed; rotenone (0.5 µM) was added to inhibit complex I and, finally, antimycin-A (2.5 µM) was added to evaluate the residual, non-mitochondrial oxygen consumption (ROX). The oxygen consumption was expressed as pmol O_2_ per second per 10^8^ cells (pmol x s^−1^ × 10^−8^ cells). All values recorded for the states of ROUTINE, LEAK and ET capacity were corrected for ROX. Moreover, to better assess the differences in mitochondrial functioning between the tested variants, the following flux control ratios were calculated: ROUTINE control ratio (*R/E*) indicating the portion of the electron transfer (ET) capacity used during routine cell activity; LEAK control ratio (*L/E*) indicating the proportion of uncoupled or dyscoupled respiration to ET capacity; and netROUTINE control ratio ((*R − L)/E*), indicating the proportion of total electron transfer capacity used for oxidative phosphorylation [41].

Measurement of respiration rates in permeabilized platelets began by capture of ROUTINE respiration, as with the intact platelets; then, substrates for CI were added to the chamber (5 mM pyruvate and 2 mM malate, PM); platelets were then permeabilized with digitonin (1 µg/10^6^ platelets). After respiration stabilized, complex I-linked LEAK state was captured, reflecting the rate of mitochondrial respiration with exogenous substrates. Following this, 1 mM of ADP was added to the chamber to evaluate CI-linked ATP production (oxidative phosphorylation, OXPHOS CI_PM_). The outer mitochondrial membrane integrity was then tested: cytochrome c was added to the chamber (10 μM) and the difference in respiration before and after cytochrome c addition was assessed. Glutamate (10 mM) was then added to evaluate phosphorylation supported by combination of pyruvate, malate and glutamate (OXPHOS CI_PMG_). Succinate (50 mM) was added to assess joint CI- and CII-linked ATP production (OXPHOS CI+CII). Maximal electron transfer capacity joint for CI and CII was checked by titration of 0.25 µM aliquots of an uncoupler (FCCP) (ET CI+CII). ETS was then assessed only for CII by inhibition of complex I with rotenone (0.5 µM) complex I inhibitor (ET CII). Finally, residual oxygen consumption (ROX) was checked after complex III inhibition by the antimycin A addition (2.5 µM). ROX was subtracted from all measured parameters. To better assess the differences in mitochondrial functioning between the tested variants, the following control ratios were calculated: OXPHOS CI+CII/ ET CI+CII, indicating the portion of the electron transfer capacity used during oxidative phosphorylation, and ET CII/ ET CI+CII, indicating the proportion of the electron transfer capacity supported by CII substrate to ET capacity supported by combination of CI and CII substrates.

### 4.7. Statistical Analysis

The data were presented as median and interquartile range (IQR) (lower (25%) to upper (75%) quartile) to easily compare numerical data between experiments and between related variables. Data normality was verified using the Shapiro–Wilk test, and homogeneity of variance was confirmed using Levene’s test. Mauchly’s test was used to test for data sphericity. Data with confirmed normal distributions and homoscedasticity/sphericity were tested with parametric tests and the remaining with non-parametric tests. Unpaired Student’s *t* test was used when comparing two independent groups with distributions not departing from normality. When normality assumption and/or variance homogeneity assumption was violated, the significance of differences was evaluated with Mann–Whitney’s U test. For multiple groups with heterogeneous variances, the non-parametric Kruskal–Wallis test and the non-parametric Conover–Inman’s post-hoc multiple comparisons test were used. The sidedness of the tests used was selected based on the null hypothesis, indicative of the expected direction of an effect. Statistical analyses were performed using Statistica v.13 (Dell Inc., Tulsa, OH, USA) and GraphPad Prism v.5 (GraphPad Software, San Diego, CA, USA).

## Figures and Tables

**Table 1:** Mitochondrial respiration parameters, control ratios and non-mitochondrial respiration measured for intact blood platelets obtained from control animals incubated with different concentrations of metformin.

Mitochondrial Parameters	Metformin Concentration					
	0 μM (Control)	20 μM	100 μM	500 μM	1000 μM	5000 μM
ROUTINE (pmol x s^−1^ × 10^−8^ cells)	21.7; 15.1–25.6	23.5; 16.9–27.3	18.4; 15.0–26.9	21.7; 17.8–23.7	14.7; 11.4–15.8 *	11.3; 9.7–14.7 **
LEAK (pmol x s^−1^ × 10^−8^ cells)	1.7; 0.9–4.1	2.7; 1.9–3.3	1.8; 0.6–2.8	1.9; 1.6–2.9	2.0; 0.9–3.2	2.3; 1.0–3.6
ET (pmol x s^−1^ × 10^−8^ cells)	23.7; 16.9–27.0	25.4; 19.7–28.8	24.0; 19.2–28.2	24.3; 21.2–25.8	16.5; 12.1–17.9 *	14.1; 12.2–17.3 **
ETrot (pmol x s^−1^ × 10^−8^ cells)	4.9; 3.4–6.5	4.7; 4.4–5.5	3.8; 2.6–4.4	4.8; 2.9–5.9	4.7; 4.2–6.2	4.6; 1.6–5.7
ROX (pmol x s^−1^ × 10^−8^ cells)	1.9; 0.8–4.4	2.5; 1.6–3.6	2.2; 1.5–5.6	2.6; 1.3–4.4	2.6; 1.0–3.5	2.5; 1.2–5.1
R/E (a.u.)	0.89; 0.86–0.96	0.92; 0.86–0.97	0.86; 0.68–0.96	0.89; 0.77–0.94	0.89; 0.83–0.92	0.79; 0.71–0.92
L/E (a.u.)	0.08; 0.04–0.19	0.10; 0.08–0.18	0.08; 0.03–0.11	0.08; 0.06–0.13	0.12; 0.06–0.21	0.16; 0.06–0.28
netR/E (a.u)	0.82; 0.74–0.88	0.80; 0.75–0.86	0.78; 0.58–0.92	0.82; 0.67–0.86	0.72; 0.70–0.84	0.66; 0.49–0.79

Data presented as medians and interquartile ranges (IQR), *n* = 8. More detailed descriptions of the measured parameters are given in Section 4.6. All values recorded for the states of ROUTINE, LEAK, ET and ETrot capacity were corrected for ROX. The significances of the differences, as estimated with the repeated measures ANOVA and the post-hoc Dunnett’s multiple comparison test, were: * *p* < 0.05, ** *p* < 0.01. Reduced ROUTINE respiration and maximal electron system capacity (ET) were noted for metformin at 1 mM and 5 mM. No significant differences were observed between platelets incubated with metformin and those not incubated (control group) for LEAK respiration, ETrot, ROX respiration or control ratios (R/E, L/E, netR/E).

**Table 2:** Activation and reactivity of rat blood platelets evaluated after two-hour incubation of isolated platelets with different metformin concentrations.

P-Selectin Expression (%)	Metformin Concentration					
	0 μM (Control)	20 μM	100 μM	500 μM	1000 μM	5000 μM
resting	8.1; 6.1–11.	8.7; 6.3–14.0	7.2; 5.3–13.2	8.8; 6.1–13.0	9.4; 5.8–13.5	10.2; 6.2–15.5
20 μM ADP	58.6; 32.3–70.7	41.3; 37.4–61.4	47.7; 32.9–54.9	64.1; 42.6–77.6	53.7; 43.4–62.5	50.4; 41.6–53.6
20 μg/mL collagen	65.6; 53.5–76.3	65.1; 50.6–72.0	57.8; 36.4–70.0	55.1; 43.1–65.0	64.3; 42.5–71.0	54.8; 47.7–59.8
0.25 U/mL thrombin	73.8; 47.8–78.4	79.2; 63.8–81.5	76.4; 63.4–81.2	73.7; 47.4–72.6	65.0; 55.9–72.6	60.3; 48.6–70.4

Data presented as medians and IQR, *n* = 8. One-sided significance of differences was estimated with the repeated measures ANOVA and post-hoc Dunnett’s multiple comparison test. No significant differences were observed between platelets incubated with metformin and those not incubated (control group) in the expression of P-selectin on the surface of both non-stimulated (resting) platelets and the cells stimulated with 20 μM ADP, 20 μg/mL collagen or 0.25 U/mL thrombin.

**Table 3:** Body weight, blood glucose level and blood glycated haemoglobin concentrations in rats with 1-month, 2.5-month and 5-month diabetes of their age-matched control groups, as well as in control and 5-month diabetic animals treated with metformin.

	1-month			2.5-month			5-month			
	C	DM		C	DM		C	DM	C+Met	DM+Met
Body	450;	300;		500;	275;		600;	325;	600;	350;
weight (g)	438–500	249–310		475–556	244–306		580–660	280–380	573–631	285–380
		***			***			***(a)		
Glucose	243;	483;		196;	498;		253;	501;	273;	473;
(mg/dl)	229–258	406–488		175–230	458–519		231–267	486–512	250–303	457–487
		***			***			***(b)		*(c)
HbA1c	21.3;	81.7;		21.6;	85.7;		24.6;	84.5;	25.0;	75.60;
(mmol/mol)	20.4–22.0	75.1–86		20.9–22.4	71.5–93.8		21.7–28.9	71.7–92.9	23.8–29.8	64.4–80.4
		***			***			***(d)		*(e)

Data presented as medians and IQR, n = 10–19. Rats with 5-month diabetes were treated with 50 mg/ kg b.w./day metformin for 4 months. The significances of the differences between diabetic animals and control group for 1-month and 2.5-month, as estimated with the unpaired Student *t* test or Mann–Whitney U test, were: *** *p* < 0.01. The significances of the differences between tested groups for 5-month, as estimated with the Kruskal–Wallis test and the Conover–Iman post hoc test for multiple comparisons, were: * *p* < 0.05, *** *p* < 0.001. The body weight was: (**a**) lowered for DM vs. control animals. The glucose level was: (**b**) elevated for DM vs. control animals; (**c**) lowered for DM+Met vs. DM. The HbA1c level was: (**d**) elevated for DM vs. control animal€(**e**) lowered for DM+Met vs. DM.

**Table 4:** Activation and reactivity of platelets from 5-month diabetic animals and their age-matched non-diabetic controls, as well as from rats with 5-month diabetes treated with metformin and the corresponding non-diabetic control animals treated with metformin.

P-Selectin Expression (%)	C	DM	C+Met	DM+Met
resting	9.1; 2.7–9.5	13.9; 6.1–21.1 # (a)	6.7; 2.7–9.0	9.1; 4.7–22.6 * (b)
5 μM ADP	70.5; 45.0–80.9	76.0; 64.8–86.4	81.3; 69.6–82.8	83.3; 72.9–88.8
20 μM ADP	81.3; 62.7–87.1	83.7; 75.4–90.5	85.3; 80.3–88.6	88.4; 81.6–93.4
5 μg/mL collagen	12.2; 6.5–27.0	16.7; 13.4–23.2	11.7; 7.4–26.6	14.1; 12.3–21.8
20 μg/mL collagen	26.4; 10.8–50.4	38.5; 27.6–45.6	23.8; 17.7–31.4	33.5; 25.5–43.7
0.025 U/mL thrombin	38.0; 14.5–43.2	36.2; 17.9–52.8	20.9; 14.0–35.1	27.3; 16.8–41.3
0.25 U/mL thrombin	93.5; 92.2–95.5	87.9; 82.0–89.3 ** (c)	95.4; 92.6–96.5	93.2; 90.1–95.3 ** (d)

Data are presented as medians and IQRs; *n* = 11–14. P-selectin expression was measured on the surface of resting and agonist-stimulated platelets (5 or 20 µM ADP, 5 or 20 µg/mL collagen, 0.025 or 0.25 U/mL thrombin) collected from rats with 5-month diabetes and their age-matched controls, as well as from 5-month diabetic and control rats treated with metformin. The significances of the differences, as estimated with the Kruskal–Wallis test and the Conover–Iman post hoc test for multiple comparisons, were: ^#^ *p* < 0.057, * *p* < 0.05, ** *p* < 0.01. For non-stimulated platelets, the P-selectin expression was: (**a**) elevated for DM vs. control; (**b**) elevated for DM+Met vs. C+Met. For platelets stimulated with 0.25 U/mL of thrombin the P-selectin expression was: (**c**) lowered for DM vs. control; (**d**) elevated for DM+Met vs. DM. No significant differences were observed between the tested groups in the expression of P-selectin on the surface of platelets stimulated with 5 or 20 μM ADP, 5 or 20 μg/mL collagen, or 0.025 U/mL thrombin.

## Data Availability

The data presented in this study are available on request from the corresponding author.
